# Results from a US modified Delphi consensus to define disease progression and disease modification in polycythemia Vera

**DOI:** 10.1007/s00277-025-06641-2

**Published:** 2025-10-15

**Authors:** Prithviraj Bose, Emily Nagler, Muhammad Sarfraz Nawaz, Raajit K. Rampal, Tsewang Tashi, Swapna Thota, John O. Mascarenhas

**Affiliations:** 1 Department of Leukemia, The University of Texas MD Anderson Cancer Center, Houston, TX USA; 2 Prebys Cancer Center, Scripps, San Diego, CA USA; 3 Wood College of Osteopathic Medicine and Hematology Oncology of Indiana, Indianapolis, IN USA; 4 Memorial Sloan Kettering Cancer Center, New York, NY USA; 5 Division of Hematology and Hematologic Malignancies, University of Utah, Salt Lake City, UT USA; 6 The University of Tennessee Health Science Center, Knoxville, TN USA; 7 Tisch Cancer Institute, Division of Hematology/Oncology Icahn School of Medicine at Mount Sinai, One Gustave L. Levy Place, 1079, New York, 10029 NY USA

**Keywords:** Polycythemia vera, Disease progression, Disease modification, United states, Delphi consensus

## Abstract

Within 15 years of diagnosis, around 15% of polycythemia vera (PV) cases progress to post-PV myelofibrosis, and around 3% transform to acute myeloid leukemia within 10 years. Risk factors for disease progression include advanced age and the presence of certain acquired myeloid-relevant mutations. A better understanding of what defines disease progression in PV is essential when evaluating treatment options that enable disease modification and improved patient outcomes. A modified-Delphi method was used to convene a virtual steering group (SG) of seven US-based clinical physicians specializing in the management and treatment of PV. The group generated 41 consensus statements across six main domains. A survey was developed from these statements and shared with a broader panel of specialists, using a four-point *Likert* scale. The results were analyzed to determine the level of agreement for each statement. The consensus threshold was set at 75% agreement. A total of 61 responses were received from hematologist-oncologists (*n* = 56), oncologists (*n* = 3), and hematologists (*n* = 2). On analysis, 39/41 (95%) statements achieved consensus agreement, and 2/41 (5%) failed to achieve consensus. Consensus statements related to response assessment adjudication, treatment selection, and disease progression scored the highest, with 92% consensus among the wider panel. Based on the consensus, the SG agreed on a set of recommendations for defining disease progression and disease modification in PV. These recommendations aim to improve understanding of the disease course to support the development of more effective therapeutic strategies.

**Supplementary Information:**

The online version contains supplementary material available at 10.1007/s00277-025-06641-2.

## Introduction

Polycythemia vera (PV) is a chronic malignancy classified within the group of Janus kinase 2 (*JAK2*) mutation-driven myeloproliferative neoplasms [1, 2]. The hallmark of PV is clonal erythrocytosis, but leukocytosis and thrombocytosis also often occur [3]. Subsequent hyperviscosity and thrombosis cause a range of symptoms related to impaired oxygen delivery, such as fatigue, headache, pruritus, microcirculatory disturbances, and an increased risk of thrombosis, which can shorten patient survival [1, 4, 5]. In around 15% of cases, PV progresses to myelofibrosis (MF) within 15 years [6]. Leukemic transformation to acute myeloid leukemia (AML) occurs in around 3% of cases within 10 years, and carries a grim prognosis with a median survival of 3–6 months from diagnosis [7, 8]. 

Deterioration of symptoms often correlates with disease progression [9]. Risk factors associated with progression include advanced age, leukocytosis, bone marrow reticulin fibrosis, splenomegaly, abnormal karyotype, and higher *JAK2V617F* variant allele frequency (VAF), *TP*53 or *RUNX*1 mutations, and the use of agents with leukemogenic properties [6]. Although the primary treatment targets center on reducing thrombotic risk, slowing disease progression (sometimes called ‘disease evolution’) is equally critical [4, 10]. While disease progression to AML or MF is well-defined, recognizing progressive disease within PV is important to enable timely intervention and improve patient outcomes [4]. 

By targeting the underlying molecular pathogenesis, certain treatment agents are considered disease-modifying, with the potential to reduce progression, improve quality of life (QoL), and extend survival [4]. Therefore, the concept of disease modification is a cornerstone of drug development, aiming to transform the treatment approach [11]. Interferon alpha (IFN-α) is considered the exemplar of a disease-modifying agent, as it can significantly and durably reduce the *JAK2* VAF [4]. In addition, the JAK inhibitor ruxolitinib (RUX) has demonstrated durable molecular responses in long-term studies [12], and several other classes of agent are currently under investigation [13].

Several additional parameters associated with disease modification have been proposed, including changes in inflammatory cytokine levels and bone marrow histopathologic markers [11]. These changes may correlate with clinically relevant outcomes, such as reduced thrombotic events, improved event-free survival (EFS), overall survival (OS), and decreased progression to MF or AML. Despite advancements, a significant unmet need remains in fully understanding disease modification in PV, and a clearer understanding could reshape treatment approaches and goals [11].

To address these gaps in the evidence base, the Delphi consensus method was employed in this study to define disease progression and disease modification in PV.

## Methods

**Fig. 1:**
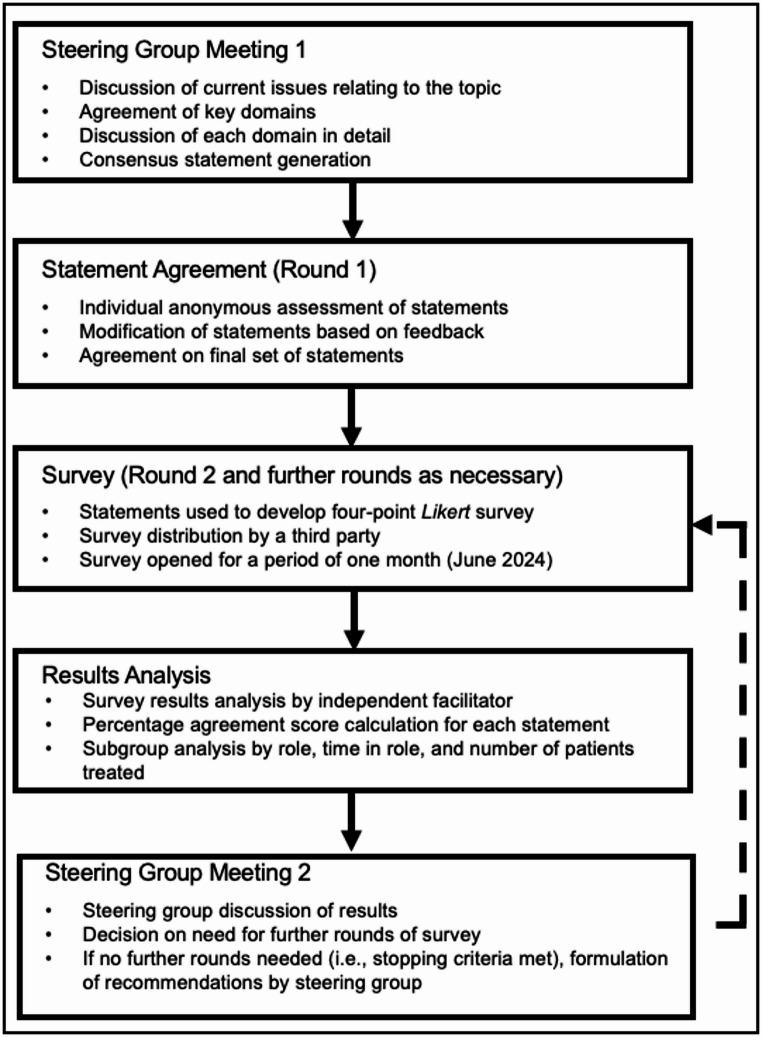
Modified Delphi study steps and objectives

A modified Delphi consensus method was employed in this study (Fig. 1). The study was not registered, and all reporting follows the ACCORD guidelines [14].

A brief review of the literature was conducted using PubMed and Google Scholar, focusing on publications from 2009 to 2024. Search terms included, but were not limited to ‘polycythemia vera’, ‘disease progression’, ‘clinical deterioration’, and ‘remission’. After review, 30 papers were selected and used to inform the agenda and stimulus for a steering group (SG) meeting.

A SG of seven US-based physicians specializing in the management of PV convened virtually in May 2024. The SG were selected based on their expertise, previous publications, years of professional experience, and research interests. During the meeting, the SG discussed the current PV treatment landscape, focusing on disease progression and disease modification, and agreed upon six domains of focus for the research:

1. Treatment goals for patients with PV.
2. Selecting the best treatment for patients with PV.
3. Defining and assessing disease modification.
4. Recognizing suboptimal response, intolerance, or resistance to treatment.
5. Defining and assessing disease progression.
6. Optimally engaging patients with PV.

These domains were discussed further, and statements were proposed by the SG working collaboratively. The initial statements were then independently reviewed after the meeting by each SG member who rated each statement as either “accept”, “remove”, or “reword with suggested changes”. Recommendations were accepted based on a simple majority. This constituted the initial round of consensus.

The final set of 41 statements were then developed for testing with a wider panel of experts. A four-point *Likert* survey was created, allowing responses such as ‘strongly disagree’, ‘tend to disagree’, ‘tend to agree’, and ‘strongly agree’. The survey was shared with a broader audience of hematology/oncology specialists by a third party (M3 Global). M3 Global were solely responsible for survey administration and raw data collection; they did not influence study design, data analysis, or results interpretation. M3 Global holds a large panel of healthcare professionals, each respondent received a nominal and universal compensation for their participation. With prior written consent, respondents were recruited according to the following criteria:

- Residence and practice in the USA.
- Specialty of hematology, oncology, or hematology-oncology.

The identities of individual respondents were not known to the SG or the independent facilitator (Triducive Partners Ltd), ensuring the anonymity of the survey. Participants were required to have experience in managing and treating patients with PV. A consent statement was included at the start of the survey.

The predefined stopping criteria were established as a minimum of 60 responses and a consensus threshold set at 75% [15]. Analysis of responses was carried out in July 2024, and the SG reconvened in November 2024 to discuss the results.

All completed surveys were analyzed to determine the overall agreement score for each statement. Based on the results, the SG derived key recommendations.

## Results

After SG review and agreement, 41 statements were included in the survey and subsequently tested. A total of 61 responses were received, and all responses were included in the final analysis. Responders were predominantly hematologists-oncologists (*n* = 56), oncologists (*n* = 3), and hematologists (*n* = 2) (Supplemental Figure A). The majority of respondents had experience of treating more than 10 patients with PV (*n* = 49) (Supplemental Figure B), and over half of the respondents had more than 10 years’ experience in their role (*n* = 39) (Supplemental Figure C).

**Fig. 2:**
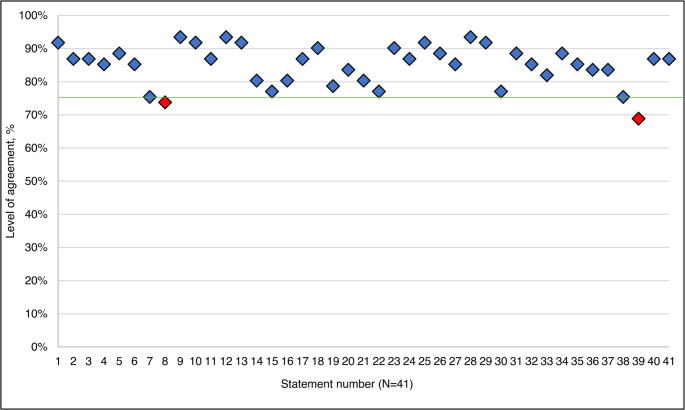
Overall agreement levels for all statements. The green line represents the 75% threshold for consensus

**Fig. 3:**
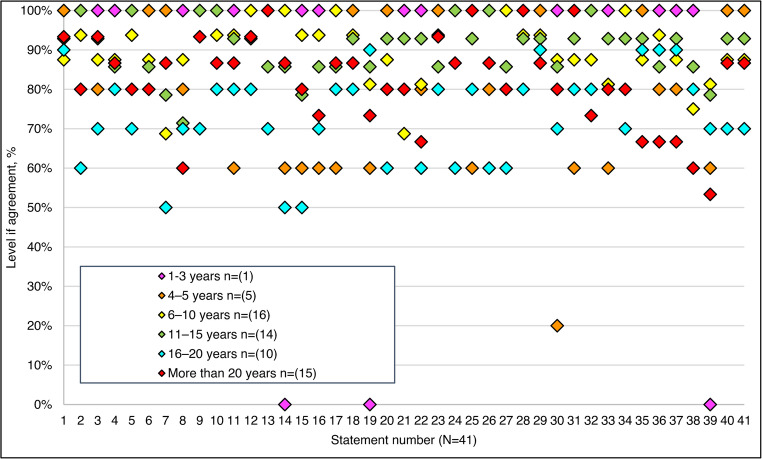
Agreement levels for all statements according to time in role

Consensus was achieved for 95% (*n* = 39/41) of the statements, and two failed to meet the threshold (Fig. 2). Consensus according to respondent declared time in role is shown in Fig. 3, and agreement according to specialist roles is represented in Supplemental Figure D.

Tables 1 and 2 contain the set of statements included in the survey along with the corresponding agreement percentages based on the four-point *Likert* scale; this is also displayed in Supplemental Figure E.

**Table 1:** Consensus agreement for each statement, domains A – C (all numbers rounded to the nearest whole number)

No:	Statement:	Strongly Agree	Tend To Agree	Tend To Disagree	Strongly Disagree	Agreement
Domain A. Treatment goals for patients with polycythemia vera (PV)						
S1	Treatment goals for PV should be prioritized according to the individual patient	54%	38%	5%	3%	**92%**
S2	Shared decision-making is important to improve outcomes in PV, including compliance and setting goals	43%	44%	8%	5%	**87%**
S3	Prevention of thrombosis and hemorrhage is a key treatment goal for PV	61%	26%	10%	3%	**87%**
S4	Prevention of progression to post-PV myelofibrosis (MF) or acute myeloid leukemia (AML) is a treatment goal for PV	46%	39%	10%	5%	**85%**
S5	Improving symptoms and maintaining good quality of life (QoL) are key treatment goals for PV	56%	33%	7%	5%	**89%**
S6	Achieving blood count control, including hematocrit, leukocytes, and platelets, is a treatment goal for patients with PV	33%	52%	10%	5%	**85%**
S7	Curing PV should be an aspirational goal	28%	48%	18%	7%	**75%**
S8	Potential expansion of drug-free ‘holiday’ with hematologic control should be an aspirational treatment goal	26%	48%	25%	2%	**74%**
**Domain B. Selecting the treatment for patients with PV**						
S9	Treatment selection should be individualized	52%	41%	7%	0%	**93%**
S10	Treatment selection should involve joint decision-making with the patient	57%	34%	8%	0%	**92%**
S11	Treatment selection should retard the progression of the disease	49%	38%	10%	3%	**87%**
S12	Comorbidity risk should be assessed prior to treatment initiation	46%	48%	7%	0%	**93%**
S13	Patients with existing multiple comorbidities (hypertension, diabetes, hyperlipidemia) at risk of cardiovascular events should be considered for more aggressive therapy beyond daily aspirin, such as cytoreductive therapies and cardiovascular modifiable risk reduction	51%	41%	7%	2%	**92%**
S14	IFN treatment should be considered for patients with low-risk and high-risk PV due to its disease-modifying potential	25%	56%	18%	2%	**80%**
S15	Phlebotomy should mainly be used as an adjunct to other treatments, such as cytoreductive therapies	31%	46%	18%	5%	**77%**
S16	New treatment options provide a better side effect profile and challenge the current paradigm of care options	38%	43%	16%	3%	**80%**
**Domain C. Defining and assessing disease modification**						
S17	Myelofibrosis-free survival can be considered as a clear survival surrogate for PV disease modification	36%	51%	10%	3%	**87%**
S18	Non-specific surrogates such as improving patients’ QoL, preventing thrombosis and disease progression, alongside correction of abnormal blood cell counts and reduction of mutant JAK2 allele burden, are useful means of assessing disease modification	46%	44%	7%	3%	**90%**
S19	A suitable measure of disease modification in PV is a percent decrease or elimination of the JAK2V617F mutant allele	26%	52%	18%	3%	**79%**
S20	A standardized PCR test to measure a decrease or elimination of the JAK2V617F mutant allele would help assess disease modification	28%	56%	16%	0%	**84%**
S21	Disease modification could mean resolution of bone marrow histology changes due to PV	38%	43%	20%	0%	**80%**
S22	Phlebotomy does not affect the natural course of the disease (i.e., does not modify the disease)	39%	38%	21%	2%	**77%**

**Table 2:** Consensus agreement for each statement, domains D – F (all numbers rounded to the nearest whole number)

No:	Statement:	Strongly Agree	Tend To Agree	Tend To Disagree	Strongly Disagree	Agreement
Domain D. Suboptimal response, intolerance, or resistance to treatment						
S23	Suboptimal response means that the patient is responding in some but not all aspects of the treatment goals	31%	59%	10%	0%	**90%**
S24	Suboptimal response should be differentiated from partial response, where blood counts improve but not to the level of complete response	41%	46%	11%	2%	**87%**
S25	Response should be assessed beyond just hematocrit control	51%	41%	8%	0%	**92%**
S26	Resistance/refers to the inability to achieve the desired therapeutic outcome after a critical period of time and at the maximum tolerated dose of the drug	39%	49%	11%	0%	**89%**
S27	The definition of intolerance/resistance should be established for treatments other than hydroxyurea, such as IFN, phlebotomy, etc.	28%	57%	10%	5%	**85%**
**Domain E. Defining and assessing disease progression**						
S28	Disease progression can be represented by a patient progressing with circulating blasts or AML or post-PV MF	43%	51%	7%	0%	**93%**
S29	Disease progression can be represented by a patient with worsening symptom score, progressively enlarging spleen, thrombosis, or major hemorrhage	62%	30%	8%	0%	**92%**
S30	Disease progression can be represented by a patient with a rising JAK2 variant allele frequency (VAF)	18%	59%	21%	2%	**77%**
S31	Decreasing Hgb can be a sign of progression to post-PV MF	36%	52%	10%	2%	**89%**
S32	Disease progression can be represented by a patient requiring progressively higher doses of their cytoreductive treatment	34%	51%	15%	0%	**85%**
S33	Disease progression can be represented by evidence of clonal evolution (the appearance of additional somatic mutations or cytogenetic abnormalities), such as ASXL1, EZH2, SRSF2, IDH1, IDH2, U2AF1, and TP53	34%	48%	16%	2%	**82%**
**Domain F. Engaging the patients with PV optimally**						
S34	Access to clinical trials in PV should be offered to eligible patients	51%	38%	10%	2%	**89%**
S35	Socioeconomic factors influence patients’ access to medicines for PV	44%	41%	11%	3%	**85%**
S36	Patients living with PV often experience anxiety	39%	44%	16%	0%	**84%**
S37	Patients with PV are interested in non-pharmacologic, holistic care options, which may extend beyond the scope of the clinician	26%	57%	15%	2%	**84%**
S38	Patients with PV who adopt non-pharmacologic, holistic care options may experience improvements in their QoL and treatment experience	25%	51%	23%	2%	**75%**
S39	There is discordance regarding treatment goals between patients and clinicians	21%	48%	30%	2%	**69%**
S40	Patient and healthcare professional education should be enhanced on regular basis	43%	44%	11%	2%	**87%**
S41	Patient-physician communication is key to improve treatment outcomes	51%	36%	10%	3%	**87%**

### Treatment goals for patients with polycythemia Vera (PV)

According to S1, treatment goals for PV should be prioritized according to the individual patient (92%) [13, 14]. Shared decision-making using a multidisciplinary team (MDT) approach was agreed as important for the effective management of PV (S2, 87%). 

Key treatment goals for PV were identified as prevention of thrombosis, hemorrhage, progression to post-PV MF or AML, improving symptoms (as indicated by a ≥ 10-point decrease in Myeloproliferative Neoplasm Symptom Assessment Form total symptom score or similar [16]), and maintaining a good QoL (S3, 87%; S4, 85%, and S5, 89%). However, as emphasized in S6 (85%), achieving blood count control, including hematocrit, leukocytes, and platelets, is a necessary step to preventing thrombosis or bleeding [9]. As per S7 (75%), respondents agreed that curing PV remains an aspirational goal.

### Selecting the best treatment for patients with PV

Treatment for PV should be individualized based on treatment goals, symptoms, and the preferences of both patients and HCPs (S9, 93% and S10, 92%). It is important to select a treatment option which exhibits both disease-modifying characteristics and also reduces elevated blood counts (S11, 87% and S14, 80%). 

Agreement for S15 (77%) indicates phlebotomy has a clear role in managing PV and reducing thrombotic risk regardless of age and in the absence of contraindications. However, phlebotomy does not reduce leukocytosis or thrombocytosis and should therefore be used as an adjunct to other treatments [1]. 

Controlling comorbid risk factors, such as hypertension, smoking, and dyslipidemia, with appropriate hematologic-directed therapy, can improve patient fitness and ability to access treatments [17]. Therefore, assessing comorbidity burden before treatment initiation is important, as it may impact the overall treatment strategy, including the decision to initiate early treatment or use cytoreductive therapies earlier than stated in guidelines (S12, 93% and S13, 92%).

Some therapies are already reshaping the current treatment landscape by demonstrating improvements in QoL, favorable safety profiles, and potentially better patient outcomes (S16, 80%). However, further evidence is required to evaluate long-term outcomes.

### Defining and assessing disease modification

Understanding disease modification in PV is crucial for drug development and formal prospective evaluation of a treatment approach [11]. Myelofibrosis-free survival (MFS) and OS can be used to indicate disease modification [18]. In addition, QoL, reduction in thrombotic events, reduced rate of progression, normalization of blood cell counts, and reduction in *JAK2* VAF can be used to assess disease modification in clinical practice (S18, 90%). The majority of respondents agreed on the routine measurement of *JAK2* VAF using standardized diagnostic tools, such as polymerase chain reaction (PCR) (S19, 79% and S20, 84%) [11]. Also, normalization of bone marrow function (which could be measured annually as part of routine monitoring [19]) may support the assessment of disease modification (S21, 80%) [11]. Agreement for S22 (77%) is perhaps lower than expected given that phlebotomy does not address the underlying disease process and, as such, does not reduce the risk of progression to myelofibrosis or AML [11].

### Suboptimal response, intolerance, or resistance to treatment

Suboptimal response to treatment is defined as achieving some but not all treatment goals (S23, 90%) and should be differentiated from partial response, where blood counts improve but not to the thresholds needed for complete response (S24, 87%, S25 92%). This difference emphasizes that treatment response should be assessed beyond hematocrit control alone. 

Approximately 25% of patients demonstrate resistance or intolerance to treatment [20]. While resistance generally refers to the inability to achieve desired therapeutic outcomes after a critical period of time at the maximum tolerated dose, these terms are best understood for hydroxyurea (HU) therapy (S26, 89%). Definitions of resistance or intolerance to other treatment modalities, such as IFN-α, are also needed (S27, 85%).

### Defining and assessing disease progression

Agreement with S30 (77%) supports the correlation between high *JAK2* VAF and disease progression. It has been shown clinically that a high peripheral blood *JAK2* VAF is a marker for clonal expansion of mutant hematopoietic stem cells and is an established risk factor for progression to post-PV MF. A *JAK2* VAF greater than 50% correlates with a higher leukocyte count, a higher absolute neutrophil count, a higher hematocrit (HCT), and a lower platelet count than one < 50% [21]. Moreover, various myeloid-related gene mutations may further influence disease phenotype and progression, underscoring the importance of genomic risk factor assessments (S33, 82%) [22]. Results from the PROUD-PV [23] study suggest that a VAF decline of 1% per month may be a useful indicator of disease modification, but regular (annual or biannual) assessment of *JAK2* VAF may be required to demonstrate this response.

### Optimally engaging patients with PV

Given the need for novel treatment options, respondents agreed that eligible patients should be offered participation in clinical trials (S34, 89%). However, socioeconomic factors, including commuting costs for patients living in rural or remote locations, can influence access to treatments and healthcare services (S35, 85%). Patients with hematologic malignancies, including PV, often experience anxiety and depression, which additionally impacts QoL and requires early support (S36, 84%) [24]. 

Non-pharmacologic, holistic care options, such as lifestyle modifications, are of interest to some patients. A Mediterranean diet rich in vitamins and minerals, maintaining a healthy weight through regular physical activity such as yoga [25], and smoking cessation, can decrease the risk of developing thromboses, comorbidities, and enhance QoL, as respondents agreed (S37, 84% and S38, 75%) [26]. 

Providing education for patients and HCPs about PV was agreed on by the respondents as important (S40, 87%), and this should include disease course understanding, symptom management, proactive prevention of adverse events, information on novel treatments available, and ongoing clinical trials. In addition, close communication between patients and HCPs is key to improving treatment outcomes (S41, 87%) [27]. 

The SG proposed the following recommendations intended to define disease progression and disease modification in PV:

1. *Alongside traditional treatment goals such as preventing thrombosis*,* controlling blood counts*,*and improving symptoms*,* preventing progression to post-PV MF or AML are key treatment goals in PV.*
2. Persistent leukocyte counts *of ≥ 15 × 10*^*9*^*/L* maintained over ≥ 5 years [28] are significantly associated with increased risk of progression to post-PV MF, myelodysplastic syndrome, or AML.
3. *Disease modification is a broad term that involves reductions in JAK2* VAF, *and may occur without complete resolution of bone marrow histology.*
4. *Reduction in JAK2 VAF is a strong indicator of disease modification which can be achieved with IFN-α or RUX treatment.*
5. *There is a need to develop a standardized and validated testing approach for JAK2* VAF. *Any testing of variant allele burden should be routinely (e.g.*,* annually) used to assess treatment response in patients receiving therapies that have the potential to reduce disease burden*,* and which can be measured by quantitative JAK2V617F using PCR techniques.*
6. *Suboptimal response in PV is evaluated based on blood cell count control*,* spleen size changes*,* and symptoms*,* considering individual patient characteristics. Intolerance refers to side effects*,* while resistance refers to failure to achieve expected outcomes.*
7. *Disease progression includes patients’ subjective symptoms and objective disease markers*,* such as increased mutant JAK2* VAF, *acquisition of new mutations*,* new or worsening splenomegaly*,* circulating blasts*,* cytopenias*,* requirement of progressively lower doses of cytoreductive therapy*,* or increased phlebotomy requirements.*
8. *There is a need for head-to-head treatment comparison trials and increased clinical trial enrollment*,* especially for patients from diverse socioeconomic backgrounds*,* to establish uniform treatment targets.*

## Discussion

This modified Delphi consensus achieved high overall agreement for all but two of the proposed statements; however, several statements did not reach uniform agreement. Variations in response demonstrate persistent areas of uncertainty in PV management, such as the prognostic relevance of persistent leukocytosis, the precise definition of disease modification, and the optimal integration of molecular monitoring. These unresolved questions align with prior reports that PV therapy still lacks consensus on several fundamental issues [11, 29]. As a consequence, these consensus statements are intended to provide a framework reflecting current opinion rather than an incontrovertible standard, acknowledging that evolving evidence and future trials may necessitate refinement of these definitions. 

Each recommendation is discussed separately below.

1. ***Alongside traditional treatment goals such as preventing thrombosis***,*** controlling blood counts***,*** and improving symptoms***,*** preventing progression to post-PV MF or AML are key treatment goals in PV.***
  PV is associated with a high disease burden, resulting in decreased QoL and survival for patients [12, 30]. Approximately 50% of patients present with PV-related symptoms at the time of diagnosis [31]. Patient treatment goals may therefore vary according to symptomology, thus addressing these goals requires effective dialogue between the patient and physician. Literature continues to debate what should be prioritized as a treatment goal. The main goals for treating PV are suggested as alleviating symptoms, improving survival, and preventing thrombohemorrhagic complications [29, 32]. In addition, it is recommended to achieve a sustained hematocrit level below 45% without phlebotomy, leukocytes under 10,000/µL, platelets under 400,000/µL, and control of splenomegaly [33]. However, this Delphi consensus study identified additional treatment goals.
  Although most symptoms improve with appropriate treatment selection, patients with active thrombosis should also receive anticoagulation treatment [33]. Documenting symptom score at the time of diagnosis and regular ongoing monitoring utilizing the same method are important.
  Risk stratification should be performed for each patient to guide treatment strategy. Well-recognized risk factors include age and thrombosis history [29, 32]. Comorbidities and body mass index should also be considered due to the impact of comorbidity burden on survival. The coexistence of neoplasms with other chronic conditions influences not only treatment decisions but also patient outcomes [17]. Previous thromboses and hypertension are significantly associated with higher thrombotic risk [34]. Therefore, an individualized treatment approach is favored.
  Although PV is considered incurable, with no current treatment completely eliminating the risk of progression to MF or AML, several case studies report remission under specific conditions [35–38]. If a deep and durable response is achieved, some therapeutic options may enable patients to take breaks from treatment while maintaining hematologic control, known as a drug-free ‘holiday’. Such holidays may occur when the *JAK2* VAF is reduced sufficiently, and while Kiladjian et al. [39] suggest a VAF of < 10% as part of the criteria for treatment discontinuation, this threshold requires validation [11].
2. ***Persistent leukocyte counts of ≥ 15 × 10***^***9***^***/L maintained over ≥ 5 years are significantly associated with increased risk of progression to Post-PV MF***,*** myelodysplastic syndrome***,*** or AML.***
  A retrospective database analysis of 520 PV patients [10] found that persistently elevated leukocyte trajectories were not associated with increased risk of a thrombotic event, but were significantly associated with increased risk of progression, and neither hematocrit nor platelet count were associated with an increased risk of either event. The greatest risk was observed in patients with a persistent leukocyte count of ≥ 35 × 10^9^/L. In addition, results from REVEAL show a leukocyte count of ≥ 11 × 10^9^/L as a predictor of progression over 3.7 years of follow-up [28].
  Results from the PV-ARC study support the lack of correlation between leukocytosis and thrombotic risk [40]. However, other studies support leukocytosis as a risk factor for thrombosis, suggesting that further work is needed to clarify the relationship. Specifically, the REVEAL study demonstrated that in patients with high-risk PV, hematocrit level >45% and leukocyte count >11 × 10^9^/L were significantly associated with increased thrombotic risk; among low-risk PV patients, leukocyte count ≥ 11 × 10^9^/L remained associated with thrombotic events [41].
3. ***Disease modification is a broad term that involves reducing JAK2 VAF and may occur without complete resolution of bone marrow histology.***
  Phlebotomy and cytoreductive therapies remain standard care options [1, 42]. However, these strategies are not associated with prolonged survival, altering the disease course, or preventing progression to MF and AML [32, 43]. Novel treatment approaches targeting underlying pathophysiological mechanisms are necessary to modify the disease’s natural course and prevent progression to MF or AML.
  A large retrospective study has validated MFS and OS as clinical endpoints, demonstrating significantly higher MFS and lower mortality with IFN-α compared to hydroxyurea (HU) and phlebotomy [18]. The disease-modifying potential of IFN-α is linked to decreased *JAK2* VAF and can be applied to patients with low-risk or high-risk PV [1, 4].
  Ruxolitinib (RUX) has also demonstrated efficacy over 8 years in reducing *JAK2* VAF from a median of 68% to 3.5% in 77 patients with PV and essential thrombocythemia (ET). Of these, 54.5% were HU-resistant, and 45.5% were HU-intolerant. Results demonstrate long-term treatment with RUX produces DMR (VAF ≤ 2%) in about 20% of PV and ET patients [12].
  Repeat bone marrow biopsy is required to assess treatment response, though it is not routinely performed [11]. According to the European LeukemiaNet 2013 criteria, complete remission involves normalization of bone marrow histology, such as the disappearance of megakaryocyte hyperplasia and the absence of reticulin fibrosis above grade 1 [16]. As novel therapies may change the natural course of the disease by reducing the *JAK2* VAF, the normalization of bone marrow histology, associated with survival and reduction in thrombosis risk, should be considered a parameter of disease modification [11].
  While this consensus primarily focused on clinical definitions of progression and modification, prior mechanistic studies have elucidated that clonal evolution, bone marrow fibrosis, and pro-inflammatory cytokine dysregulation play key roles in PV disease course [1, 44, 45], supporting the need to integrate molecular correlates with clinical criteria.
4. ***Reduction in JAK2 VAF is a strong indicator of disease modification which can be achieved with IFN-α or RUX treatment.***
  Reduction in *JAK2* VAF is increasingly becoming an important component of PV treatment and a potential marker for disease modification, linked to lower venous thrombosis and reduced disease progression. However, data from REVEAL showed that, at the time of diagnosis, < 50% underwent *JAK2* mutation testing in the US, and of those who did undergo *JAK2* mutation testing, approximately 96% had a *JAK2* V617F mutation [31]. Although this may have improved in the years since publication, clinicians should ensure that *JAK2* mutation testing is performed in a timely manner for the appropriate use of mutation-targeted therapies. RCT evidence supports the correlation between durable molecular response and event-free survival (EFS) in PV. The MAJIC-PV [46] study found that those who achieved a 50% reduction in *JAK2* VAF at 12 months were more likely to demonstrate complete response at 12 months (*P* = 0.09). In addition, the PROUD-PV and CONTUNATION-PV [4, 23] studies found that molecular responses to treatment were accompanied by improved EFS.
  A baseline *JAK2* VAF < 50%, and a VAF reduction of ≥ 35% after 2 years of treatment, have been found to be predictive of achieving a durable molecular response and reduced progression to myelofibrosis [12], further supporting the use of *JAK2* VAF (and surrogates) in determining molecular response and disease modification.
  The role of Neutrophil-to-Lymphocyte ratio (NLR) is growing as a surrogate for JAK2V617F suppression, as reported by Barbui et al. [47] This analysis of data from ECLAP, Low-PV, and PROUD-PV/CONTINUATION-PV found a strong correlation between reduction and *JAK2* VAF and NLR reduction. Individuals who experienced >50% NLR reduction also demonstrated a −59.3% change in *JAK2* VAF, and those minimal NLR change had only a −13% corresponding reduction in *JAK2* VAF. This supports the use of NLR as a surrogate indicator of disease modification. And, while both IFN-α and HU treatment were found to reduce leukocytosis and neutrophilia, IFN-α was also shown to normalize NLR, lower *JAK2* VAF, and better preserve lymphopoiesis. [47]
  In terms of outcomes, a prospective cohort analysis found that patients with PV and an NLR ≥ 5 had significantly worse overall survival and more than twice the mortality rate compared to those with NLR < 5 [48]. In analysis of data from the ECLAP study, Carobbio et al. found that the relative risk of venous thrombosis was almost doubled in individuals with an NLR ≥ 5 [49]. Furthermore, investigation into the effect of IFN-α vs. phlebotomy-only on NLR found that not only was IFN-α superior in reducing NLR (*p* = 0.008), but that reduction of NLR was linearly associated with reduction in *JAK2* VAF. This suggests that in practice, a decreasing NLR could have clinical utility as an easy way to measure indicator of treatment success over time [50].
5. ***There is a need to develop a standardized and validated testing approach for JAK2*** **VAF**, ***which should be routinely used to assess treatment response.***
  We stress the importance of *JAK2* VAF reduction and its associations with improved EFS, but this is not routinely measured or included in treatment decision-making. Use of next-generation sequencing (NGS) or PCR should be routine in assessing treatment response to those therapies that have the potential to reduce disease burden. A standardized and validated approach to *JAK2* VAF testing should be agreed and implemented, as has been successfully done for BCR-ABL1 in chronic myeloid leukemia.
6. ***Suboptimal response in PV is evaluated based on cell count control***,*** spleen size changes***,*** and symptoms***,*** considering individual patient characteristics. Intolerance refers to side effects***,*** while resistance refers to failure to achieve expected outcomes.***
  Approximately 30–40% of patients with PV demonstrate suboptimal responses to cytoreductive therapy [51]. In HU-refractory/intolerant PV, ruxolitinib (a JAK1/2 inhibitor) has demonstrated efficacy in achieving hematocrit/spleen and symptom control [1].
  According to the European LeukemiaNet criteria, platelet and white blood cell counts, along with disease-related symptoms, should be assessed to evaluate treatment response [16]. Since ‘resistance’ and ‘intolerance’ have already been defined for HU, this Delphi consensus proposes general definitions for ‘resistance’, ‘intolerance’ and ‘suboptimal response’.
7. ***Disease progression includes patients’ subjective symptoms and objective disease markers***,*** such as increased JAK2 VAF***,*** clonal evolution***,*** new or worsening splenomegaly***,*** circulating blasts***,*** cytopenias***,*** requirement of progressively lower doses of cytoreductive therapy***,*** or increased phlebotomy requirements.***
  This consensus suggests that the above factors should be considered when assessing risk of disease progression [22]. A growing body of evidence supports the link between high *JAK2* VAF and increased risk of transformation to AML or MF, and thrombotic events [52–57]. Despite advancements in PV therapies, symptoms and clinical parameters require careful monitoring to support the assessment of disease progression and inform treatment approach. Therapeutic agents capable of altering disease course and preventing progression are necessary [22].
8. ***There is a need for retrospective real-world evidence (ideally based on registry data)***,*** especially for patients from diverse socioeconomic backgrounds***,*** to establish uniform treatment targets.***
  Patients and HCPs may have a different focus on the outcomes of PV and associated management. The MPN Landmark survey [58] reported that patients consider the most important treatment goals to be to ‘Slow/delay progression of PV’ and ‘Prevention of vascular/thrombotic events’ with maintaining hematocrit levels < 45% being the least important. In contrast, treatment goals stated in the medical literature are to reduce the risk of thrombosis and to alleviate symptoms by maintaining hematocrit at < 45% [59]. This suggests that there is a need for patients and HCPs to discuss goals and treatments and consider how patient preferences can be incorporated into the treatment approach.
  Current treatments primarily adhere to guideline-defined standards. The limited availability of clinical trials, particularly in community settings, poses challenges for patient participation, especially for patients with diverse socioeconomic backgrounds [24].

### Strengths and limitations

Strengths of this study include a wide representation of experts with extensive experience in the treatment and management of PV from across the USA and a strong concordance in results between roles. A significant number of hematologist-oncologist respondents suggests broad knowledge of the issues covered. However, high levels of agreement achieved over one round of wider testing may suggest bias in that the statements were designed to be broadly agreeable and did not sufficiently challenge the status quo. In addition, one respondent reported 1–3 years’ experience, which may not reflect sufficient seniority, however, it is not expected that this affected the agreement levels of the results.

## Conclusion

Key treatment goals in PV include preventing thrombosis, controlling blood counts, improving symptoms, and avoiding progression to post-PV MF or AML. Disease modification focuses on reducing driver mutation clone size, particularly the *JAK2* VAF, which serves as a strong marker of treatment response. Disease progression is assessed through symptoms and markers like increased *JAK2* VAF, clonal evolution, splenomegaly, circulating blasts, cytopenias, reduced response to therapy, or higher phlebotomy needs. There is a need for clinical trials, including head-to-head treatment comparisons and greater representation of diverse populations, to establish standardized treatment targets.

## Supplementary Information

Below is the link to the electronic supplementary material.

Supplementary Material 1

## Data Availability

Data is available on reasonable request from the corresponding author and Triducive Partners Ltd.

## Declarations

### Trial registration

This consensus was not prospectively registered.
