# Supplementary material for: Results from a US modified Delphi consensus to define disease progression and disease modification in polycythemia Vera
Source: Ann Hematol. 2025 Oct 15;104(10):5063–75. doi: 10.1007/s00277-025-06641-2 (PMC12619767; doi:10.1007/s00277-025-06641-2)
Supplement: Supplementary file 1 — Supplementary Material 1 [file 277_2025_6641_MOESM1_ESM.docx]

**Results from a US modified Delphi consensus to define disease progression and disease modification in polycythemia vera**

Prithviraj Bose^1^, Emily Nagler^2^, Muhammad Sarfraz Nawaz^3^, Raajit K Rampal^4^, Tsewang Tashi^5^,
Swapna Thota^6^, John O Mascarenhas ^7^

1. Department of Leukemia, The University of Texas MD Anderson Cancer Center, TX, USA
2. Prebys Cancer Center, Scripps, San Diego, CA, USA
3. Wood College of Osteopathic Medicine and Hematology Oncology of Indiana, IN, USA
4. Memorial Sloan Kettering Cancer Center, New York, NY, USA
5. Division of Hematology and Hematologic Malignancies, University of Utah, UT, USA
6. The University of Tennessee Health Science Center, TN, USA
7. Tisch Cancer Institute, Icahn School of Medicine; Mount Sinai, New York, NY, USA

**CORRESPONDING AUTHOR:** John O Mascarenhas, MD, Professor of Medicine Tisch Cancer Institute, Division of Hematology/Oncology Icahn School of Medicine at Mount Sinai, One Gustave L. Levy Place, Box 1079 New York, NY 10029, ORCID ID: [0000-0002-8400-0483](https://orcid.org/0000-0002-8400-0483), [john.mascarenhas@mssm.edu](mailto:john.mascarenhas@mssm.edu)

**SUPPLEMENTARY INFORMATION**

**Supplementary Figure A.** Respondent roles.

**Supplementary Figure B.**  Number of patients treated by respondents.

**Supplementary Figure C.** Duration of professional experience of respondents.

**Supplementary Figure D.** Agreement level according to specialist roles.

**Supplementary Figure E.** Agreement level according to each statement.
